# An Educational News Dataset for Recommender Systems

**DOI:** 10.1007/978-3-030-65965-3_39

**Published:** 2020-12-09

**Authors:** Yujie Xing, Itishree Mohallick, Jon Atle Gulla, Özlem Özgöbek, Lemei Zhang

**Affiliations:** 5 University of Sydney, Sydney, NSW Australia; 6 Monash University, Clayton, VIC Australia; 7 University of Bari Aldo Moro, Bari, Italy; 8 University of Bari Aldo Moro, Bari, Italy; 9 University of Guelph, Guelph, ON Canada; 10 University of Caen Normandy, Caen, France; 11 University of Pisa, Pisa, Italy; 12 Norwegian University of Science and Technology, Trondheim, Norway; 13 University of Porto, Porto, Portugal; 14 UPC BarcelonaTech, Barcelona, Spain; 15 University of Porto, Porto, Portugal; 16 Fraunhofer IAIS, St. Augustin, Germany; 17 Royal Holloway University of London, Egham, UK; 18 University of Lisbon, Lisbon, Portugal; 19 University of Bari Aldo Moro, Bari, Italy; 20 University of Lisbon, Lisbon, Portugal; 21 University of Bari Aldo Moro, Bari, Italy; 22 ICAR-CNR, Rende, Italy; 23 University of Naples Federico II, Naples, Italy; 24 University of North Carolina, Charlotte, NC USA; 25 Australian National University, Canberra, ACT Australia; 26 Leibniz University Hannover, Hannover, Germany; 27 Technical University of Dortmund, Dortmund, Germany; 28 University of Southern Denmark, Odense, Denmark; 29 University of Pisa, Pisa, Italy; 30 Warsaw University of Technology, Warsaw, Poland; 31 ISTI-CNR, PISA, Italy; 32 Berlin Institute of Technology, Berlin, Germany; 33 Berlin Institute of Technology, Berlin, Germany; 34 Norwegian University of Science and Technology, Trondheim, Norway; Norwegian University of Science and Technology, 7491 Trondheim, Norway

**Keywords:** Refined datasets, News recommender system, Machine learning

## Abstract

Datasets are an integral part of contemporary research on recommender systems. However, few datasets are available for conventional recommender systems and even very limited datasets are available when it comes to contextualized (time and location-dependent) News Recommender Systems. In this paper, we introduce an educational news dataset for recommender systems. This dataset is the refined version of the earlier published Adressa dataset and intends to support the university students in the educational purpose. We discuss the structure and purpose of the refined dataset in this paper.

## Introduction

The proliferation of online news creates the need for filtering and recommending specific news to focus on interesting articles. The past decade has seen a tremendous increase in the popularity of news recommender systems [1, 2]. Therefore, many online media houses have deployed news recommender systems for identifying interesting stories for its online readers and operates over a set of unlimited news sources.

With the ubiquity of access to instant news on online sources, the preference of users has changed over time from the traditional model of publishing news—from printed newspapers to online news. A large amount of news content availability on the web causes the information overload problem for online news readers. News recommender systems help these online readers in alleviating their effort in terms of time and choice by providing personalized lists of news articles. However, it is challenging for the media houses/online sources to recommend real-time news without any explicit user ratings from the users. Users are often seen moving from one news outlet to others for getting the most recent and relevant news [3]. Although controversies like filter bubbles and echo chambers remain associated with the recommender systems, most news readers prefer personalization features on news sites.

However, news recommender systems must deal with long-term user preferences and short-term trends. For instance, the long-term preference is driven by the professional activity of users, education, etc. and is best captured with content-based filtering methods. While the short-term user preference, such as popular stories, is captured using collaborative filtering methods in news recommender systems. The collaborative filtering approach tries to predict the utility of news articles for a particular user based on the news articles interacted with by other users in the past. In contrast, the content-based filtering approach tries to predict the utility of news articles for a particular user based on the new´s articles content in the past. More recent news recommender systems tend to be hybrid solutions, in which collaborative filtering and content-based recommendation are combined for news personalization.

News recommendation has a substantial practical relevance due to the specific challenges the news domain entails. Research in this area is still growing as the number of research papers increase since the last decades. The pioneers in the field of online news recommendations such as Google news have been using advanced recommendation strategies (novel scalable algorithms) for generating successful personalized news recommendation for the users of Google news [1, 2]. In recent decades, the traditional printed media like BBC, Washington Post, and New York times [4] have adopted news personalization as an attempted antidote for information overload. In Norway, the third- largest media house, Polaris media has deployed personalization features on its fully personalized first mobile news site “*iTromsø*”. A significant growth-rates in both unique visitors and pageviews have been observed. In addition, readers spent 15% more time on reading recommended articles than regular articles, and there is a 28% increase in time spent on the front page of *iTromsø* [5].

The challenges associated with the news recommender systems are different from the conventional recommender systems. For instance, the unstructured format of the news stories, recency aspects, short item life time, large volume of available news, heterogeneous nature of the information sources, greater item churn, unavailability of user rating, unique user interaction style are some of the identified challenges in case of news recommender systems [6]. A recent survey paper reveals that [7] more researchers used a hybrid approach and combined content-based filtering and collaborative filtering methods for recommending news to overcome the above said challenges.

Methodologically this approach makes use of many techniques from information retrieval, like linguistic preprocessing of content data [8] and search queries expanded with profile models [9, 10]. Experiments indicate that users also appreciate additional strategies that boost fresh news, popular news and news that take place in their own neighborhood [11]. An earlier research paper demonstrates the architecture of an advanced news recommender system. It introduces the Adressa compact data set published within the RecTech Project at the Norwegian University of Science and Technology [12]. The paper later discussed how the Adressa dataset can be used in advanced news recommender systems. Research datasets are essential for training and evaluation to accommodate various recommendation strategies. For instance, Kaggle Dataset is used in the field of predictive modeling and machine learning whereas Sage research methods dataset is used for supporting in teaching and learning data analysis techniques [3]. As a follow-up research for the earlier compact Adressa dataset, this paper introduces the refined Adressa dataset. The refined dataset is different from the previously released dataset and can be utilized for the teaching/learning activities related to news recommendation in the university setting. The refined Adressa dataset is cleaner as a substantial amount of noise is reduced and requires less preprocessing time. This dataset is suitable for educational purposes because the students need less time to preprocess the raw news data.

This paper is organized as follows. In Sect. 2 we briefly discuss relevant datasets for news recommender systems. We present the structure of the Adressa refined dataset in Sect. 3. In Sect. 4 we show how the fields should be interpreted and used followed by the conclusions in Sect. 5.

## Related Work

Evaluating recommender systems is an intricate issue and primarily recommender systems are evaluated using one of these three approaches: offline experimentation and simulation based on historical data, laboratory studies, or A/B (field) tests on real-world websites [7]. However, the research from the aforesaid paper states that offline evaluation approach is primarily used for evaluating news recommender systems as online studies are often difficult to carry out.

The types of evaluation in a recommendation setting is dependent on publicly available datasets (i.e., their size or the amount of user and item information). A dataset in this context is defined as collection of data that is used to train and test new systems under development. Some of the most used and recently published research dataset are as follows:

- *Yahoo’s datasets* are specifically tailored for unbiased offline evaluation [14] and are used in several research activities concerning news recommendation. One of the datasets, Yahoo! Front Page, comprises clicks data of two weeks from the main page of Yahoo! News. Each visit to the page was described by a binary vector of features. The 182-item pool for recommendations always contains 20 items. The log consists of nearly 28M visits to a total of 653 items. Due to the limitation of the data collection period, research is piratically not possible for personalization based long term user models.
- Swiss dataset [15] comprises of the data from the websites of two daily SwissFrench newspapers called Tribune de Gen`eve (TDG) and 24 Heures (24H) from Nov. 2008 until May 2009. The aforesaid news sites contain news stories ranging from local news, national and international events, sports to culture and entertainment. The dataset contains all the news stories displayed and all the visits by anonymous users within the time period. Each time a user browses the website, a new visit is created even if she browsed the website before.
- *SmartMedia Adressa dataset* has recently been released [7, 12] which contains click logs of approximately 20 million-page visits from a Norwegian news portal as well as a sub-sample with 2.7 million clicks (referred to as “light version”). The dataset also contains some contextual information such as geographical location, time spent on reading an article and session boundaries for the users. The data set is published with the collaboration of Norwegian University of Science and Technology, Norway and Adresseavisen as part of the RecTech Project. The dataset is collected during a span of 10 weeks (from 1 January 2017 to 31 March 2017) and contains the click events of about 2 million users and about 13 thousand articles.

Details of datasets for recommendation such as Outbrain dataset, The Plista Dataset, The Netflix dataset, Movielens dataset are addressed in the research papers [7, 15, 16] where some of the datasets like Netflix and Movielens are used to develop solution concerning collaborative filtering. There are only a few datasets available publicly for the news recommendation such as Yahoo! dataset, Plista dataset, Adressa Dataset, Kaggle dataset from Globo.com, a news portal from Brazil [13]. Extensive use of proprietary and non-public datasets in news recommendation is addressed in [2, 15] while investigating the offline performance and online success of any news recommender systems. Offline performance in this context is measured in terms of accuracy metrics whereas the online success is measured in terms of click-through-rates. These non-public datasets such as Movielens and Netflix dataset are different from the conventional dataset due to sparsity aspect as there is no cold start problem associated. Therefore, the application of such datasets in the news domain is debatable [16].

## Structure of Adressa Refined Dataset^1^

The Cxense platform^2^, the recommendation platform provided by our partner Cxense for news recommendation and monitoring, was used to extract the dataset, which covers one week of web traffic from February 2017 on the www.adresseavisen.no web site. The details of the platform can be found in the earlier paper [12]. From the raw data extracted from the Cxense platform, we construct a refined dataset that contains reading events with 9 selected attributes. The three attributes–event ID, user ID, and document ID–give the index for each event. The remaining 6 attributes offer the most important information about the reading event.

The refined dataset includes anonymized user data from the local digital newspaper from 01.01.2017 to 31.03.2017 (3 months in total). To reduce sparsity, we filter 1000 most active users from the original dataset and select 9 attributes that we think most relevant for the project. The attributes of the event table are listed in Table 1. Each reading event is given a unique ID, and the user (ID) and document (ID) that appear in these specific events are recorded, as well as the access time of the event. For each user, except for a unique user ID, the dataset also provides the active time during which the user spends on each document. For each document, the dataset provides the document ID, the title, the category, and the publish date. Also, the web page URL that the user visits (canonicalURL) is recorded.

**Table 1.** Fields of the refined adressa dataset.

Attribute	Description	Example
eventID	Id of Reading event	1082287123 (integer)
time	The time of the event	1487572383 (Unix time)
activeTime	The active time spent on a page	23 (s)
canonicalURL	URL of the visited page	“http://adressa.no”
documentID	Internal ID of page	“9757814edc2d346dfcf6f54e349f404c4e9775cf”
title	Title of the article	“Test av 19 grovbrød”
category	News category	“sport”
publishTime	Date of publication	“2017-02-20T09:45:47.000Z”
userID	The cross-site user identifier	“cx:i8i85z793m9j4yy0:cv8ghy3v45j8”

There is no explicit rating of news stories, but there are implicit signals of interests in terms of click counts and time spent reading the articles that may be used to calculate scores.

As shown at the end of the table in Table 2, the refined Adressa dataset contains 20,344 news articles, 1,000 readers, and about 700 thousand events. Each of these events corresponds to a user reading a particular news article.

**Table 2.** Comparison of some well-known datasets.

Datasets	Items	Users	Ratings	Density (%)	Rating Scale
MovieLens 1 M	3,883 movies	6,040	1,000,209	4.26	[1–5]
MovieLens 10 M	10,682 movies	71,567	10,000,054	1.31	[1–5]
MovieLens 20 M	27,278 movies	138,493	20,000,263	0.53	[1–5]
Netflix	17,770 movies	480,189	100,480,507	1.18	[1–5]
MoviePilot	25,058 movies	105,137	4,544,409	0.17	[1–5]
Last.fm 360 K	294,015 artists	359,347	17,559,530	0.017	[1, 5]
Yahoo Music	624,961	1,000,990	262,810,175	0.042	[1, 5]
Jester	150 jokes	124,113	5,865,235	31.5	[−10, 10]
Book-crossing	271,379 books	92,107	1,031,175	0.004	[1, 10] + implicit
YOW	5,921 articles	28	10010	6.0	[1, 5] + implicit
Plista	70,353 articles	14,897,978	84,210,795	0.008	Click counts
Adressa 2 M compact	923 articles	15,514	2,717,915	0.19	Click counts, reading times
Refined Adressa	20344 articles	1,000	788,931	3.34	Click counts, reading times

As seen from Table 3, for the refined Adressa dataset, Nyheter (News) make up about 48% of the articles included in the dataset. There are also many Pluss (Paid content) and sports articles in the dataset. The total number of articles which has a category field is 12,748 out of 20,344 since some articles miss category inputs.

**Table 3.** Number of articles per news category for refined dataset.

Category	No. of articles	% of articles
“Nyheter” (news)	6,169	48.39
“Pluss” (paid content)	3,106	21.38
“100sport” (100sport)	2,726	24.36
“Meninger” (opinions)	240	1.88
“Bolig” (housing)	233	1.83
“Kultur” (culture)	104	0.82
“Forbruker” (consumer)	71	0.56
“Sport” (sport)	28	0.22
“Tema” (theme)	24	0.20
“Migration catalog”	21	0.16
“Tjenester” (services)	18	0.14
“Været” (weather)	4	0.03
“Bil” (car)	2	0.02
“omadresseavisen” (about Adresseavisen)	1	0.01
*Average per category*	910.6	7.1

**Fig. 1.**
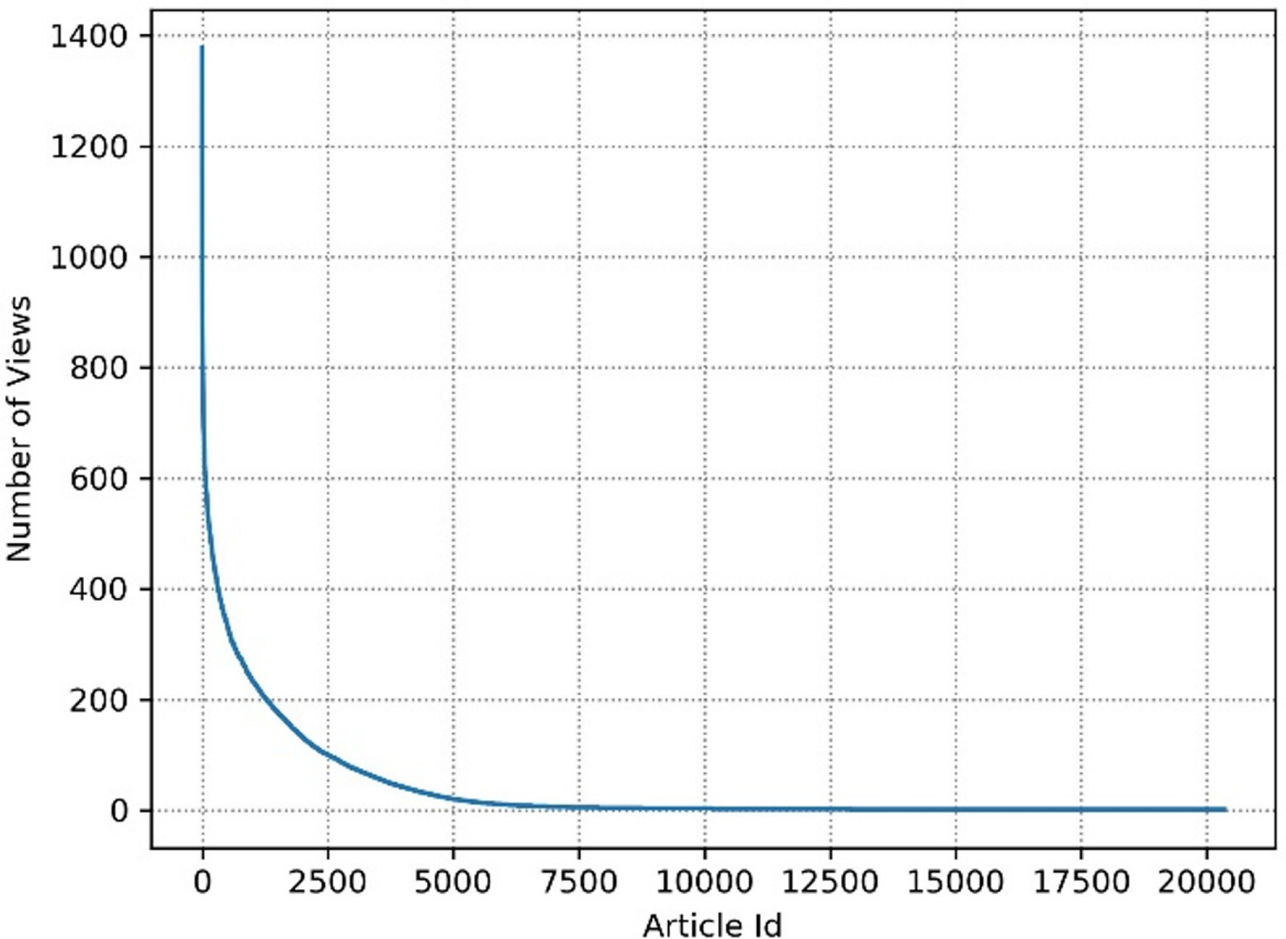
Number of article views per article for refined dataset.

In Fig. 1, we see how often the 20344 articles have been viewed by the users. Different from the compact dataset, a majority of articles-12906 articles (63.44%)-are viewed more than one time, and 2492 articles (12,25%) are viewed more than 100 times. This indicates that the refined dataset is less sparse.

Table 3 and Fig. 1 indicate that the refined dataset is balanced on the article views. More than half of the articles have been viewed more than one time, and about 10% of articles are popular (viewed more than 100 times).

## Dataset for Education

The refined dataset can be provided to students who take the recommender system course as a group project. The goal of the project is to recommend news to the users in the refined dataset, given the clicked documents, active time, publish time, access time, etc. The result is evaluated based on whether the recommended documents were clicked by the user through hit rate, click through rate, ARHR, and MSE. Students have the opportunity to compare results containing or not containing missing data, and to compare different methods of evaluation, which enables them a realistic prospective into the problem.

According to the students’ feedback, the refined dataset is more suitable for a group project lasting one semester, since the data has a much higher density than the compact dataset. Previously, the students spent lots of time on filtering the dataset, while after changing to the refined dataset, students can spend more time on analyzing the dataset and implementing more algorithms. We introduce the different algorithms that the students can implement in the following subsections.

### Collaborative Filtering

Collaborative Filtering (CF) is a widely adopted recommendation algorithm, and also an important part of the recommender system course. The fundamental assumption of CF is that if user X and Y rate n items similarly, or have similar behaviors (such as buying, rating, clicking, listening), and hence will rate or act on other items similarly. Given the access status and the active time, students can practice implementing collaborative filtering algorithms. We provide the Explicit Matrix Factorization (MF) as an example to the students.

### Content-Based Recommendation

Content-based recommendation is another popularly used recommendation method. It makes recommendations by analyzing the content of textual information and finding regularities in the content. We provide the titles and categories of documents for students to practice content-based recommendation algorithms. The example codes that we offer adopt TF-IDF (Term Frequency – Inverse Document Frequency) for feature selection and Cosine similarity to find the most similar items with user clicking before.

### Other Recommendation Algorithms

There are plenty of possibilities for students to create other algorithms. For example, they can use publish time and access time to implement temporal recommendation systems; or from collaborative filtering and content-based recommendation, they can create many different kinds of hybrid recommendation systems. Further, to improve performance, it is natural for them to utilize new technologies like deep learning.

## Discussion and Conclusion

In this paper, we introduce a refined dataset from the original Adressa dataset for training and evaluating recommender systems for news. The refined dataset contains selected users with high activity rates, and it has a much higher density than the previous dataset. We kept 9 most important attributes in the dataset.

This smaller but denser dataset is suitable for teaching. It was provided to students of recommender system courses for their course project for several years. In the future, we look forward to seeing more utility of it on education or research.

## Footnotes

1 The refined dataset can be downloaded using this link: http://Reclab.Idi.Ntnu.no/Active1000.Zip.

2 https://www.cxense.com/.
